# Strong electric wave response derived from the hybrid of lotus roots-like composites with tunable permittivity

**DOI:** 10.1038/s41598-017-09985-6

**Published:** 2017-08-25

**Authors:** Xiaohui Liang, Bin Quan, Jiabin Chen, Dongming Tang, Baoshan Zhang, Guangbin Ji

**Affiliations:** 1 School of Electronic Science and Engineering, Nanjing University, Nanjing, 210093 P. R. China; 2 College of Material Science and Technology, Nanjing University of Aeronautics and Astronautics, Nanjing, 210016 P. R. China

## Abstract

Lotus roots-like NiO/NiCo_2_O_4_ hybrids derived from Metal-organic frameworks (MOFs) are fabricated for the first time by using flake NiCo-MOF precursors as reactant templates. It was found that a thin sample consisting of 60 wt % NiO/NiCo_2_O_4_ hybrids in the wax matrix exhibited an effective microwave absorption bandwidth of 4.2 GHz at the thickness of 1.6 mm. The highest reflection loss of −47 dB was observed at 13.4 GHz for a sample with a thickness of 1.7 mm. Results obtained in this study indicate that hybrids of NiO and NiCo_2_O_4_ are promising microwave absorbing materials with adjustable permittivity, which can exhibit broad effective absorption bandwidth at low filler loading and thin thickness.

## Introduction

In the past years, the research on microwave absorbing materials has focused on low-dimensional nanomaterials. Nanomaterials are expected to provide additional interfaces and anisotropy effect, which are in favor of the attenuation for electromagnetic waves. Recent progress demonstrated that the morphology of nanomaterials has profound effects on their microwave absorption performance by changing their electromagnetic parameters in microwave range^1–3^. It is therefore of great significance to survey the morphology dependent microwave absorption properties of nanomaterials towards the design and fabrication of novel microwave absorbents.

NiCo_2_O_4_, a binary metal oxide with spinel structure, is attracted much attention in the fields of electrochemical energy storage^4–7^, solar cells^8^ and transparent conductive flms^9, 10^. Interestingly, recent research showed that the electrical conductivity of NiCo_2_O_4_ nanoplate is as high as 62 S cm^−1^ ^11^, which is at least two orders of magnitude higher than NiO and Co_3_O_4_. Moreover, efforts have been made recently to investigate the microwave absorption of nickel oxide and cobalt oxides. For example, Sun *et al*. found the microwave absorption of CoO nanobelts is stronger than that of submicrometer spheres^12^, a similar result with morphology-dependent microwave absorption properties in CoO nanostructures was also reported by Che’s group^13^. Besides, graphene was often introduced into NiO and Co_3_O_4_ nanostructures, to obtain enhanced microwave absorption due to their relatively low dielectric loss in microwave range^14–17^. Very recently, mesoporous NiCo_2_O_4_ microfiber was demonstrated by Zhang and co-workers as a promising candidate for a microwave absorbent^18^. Yet very limited progress has been made towards a fundamental understanding of the microwave loss mechanism in NiCo_2_O_4_ nanomaterial. Nevertheless, metal-organic frameworks (MOFs), as a new class of porous materials built up with metal ions/clusters and organic ligands^19^, have become more and more attractive in a variety of potential applications for their exceptional tunable porosities along with good structural robustness and flexibility^20^, such as gas storage^21^, gas separation^22^, catalysis^23^, drug delivery^24^, and energy storage^25–27^. In addition, Li and his coworkers synthesized Cz-MOF-253-supported Pd nanoparticles (Pd/CzMOF-253–800), which showed excellent performance in a one-pot sequential Knoevenagel condensation-hydrogenation reaction^28^. Jiao *et al*. designed sandwich-type metal–organic framework/graphene oxide as a template and precursor, then a layered CoP/reduced graphene oxide (rGO) composite has been successfully prepared via a pyrolysis and subsequent phosphating process^29^. Especially, MOF precursors derived uniquely porous nanoarchitectures (NiO/NiCo_2_O_4_ lotus root-like nanoflakes derived from NiCo-MOFs) is never reported so far.

In the present work, porous NiO/NiCo_2_O_4_ lotus root-like nanoflakes derived from NiCo-MOFs are fabricated via directly carbonizing the flake structured NiCo-MOFs precursor under high temperature. With the filler loading of NiO/NiCo_2_O_4_ hybrid 60 wt % in a wax matrix, the composites under the thicknesses of 1.6 mm exhibited an effective electromagnetic wave absorption bandwidth of 4.2 GHz. With the thickness of 1.7 mm, the highest reflection loss reaches −47 dB at 13.4 GHz. These results indicate that NiO/NiCo_2_O_4_ hybrid is a promising electromagnetic wave absorbing material, which can exhibit broad effective absorption bandwidth at low filler loading and thin thickness.

## Results

**Figure 1:**
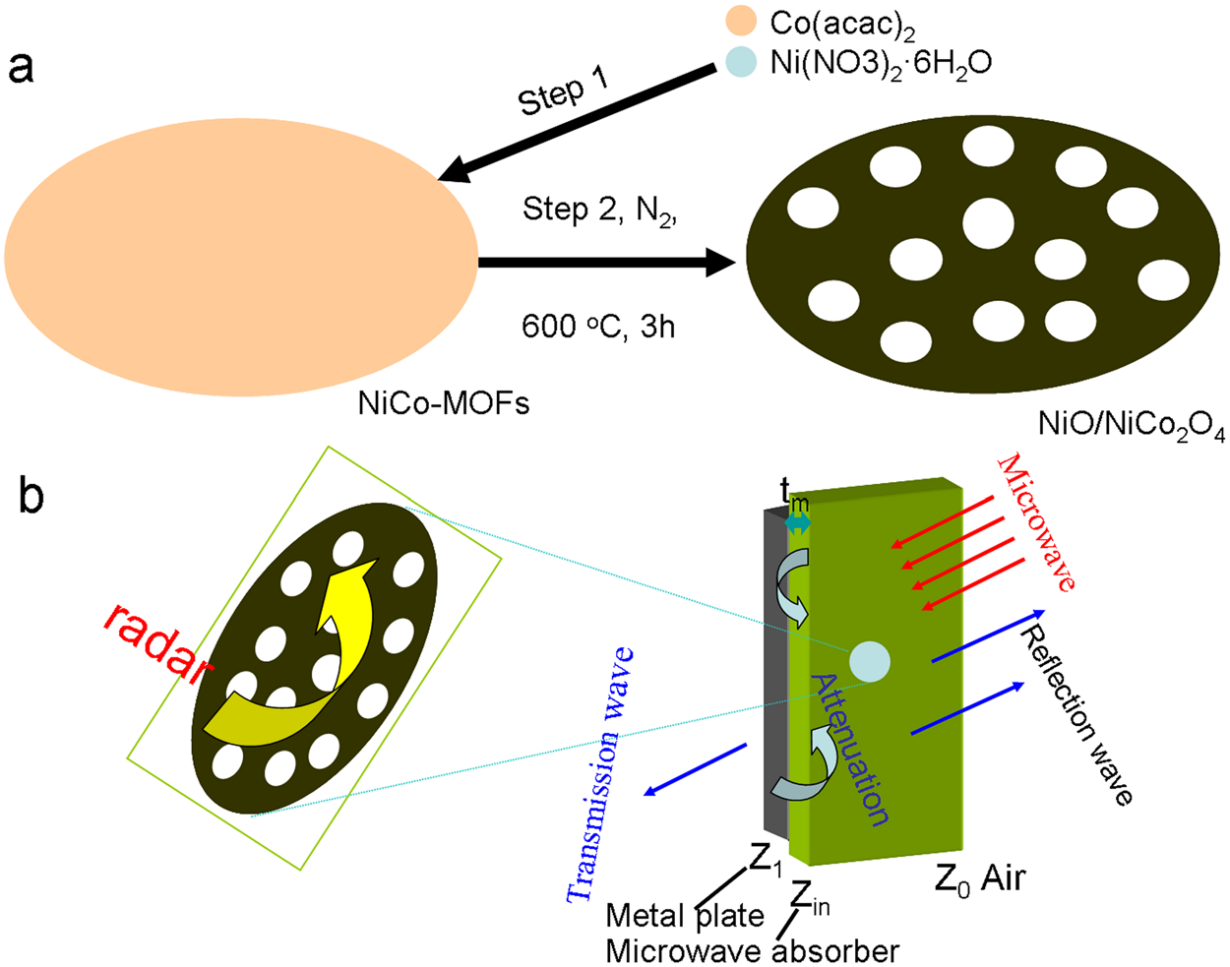
Schematic illustration of (**a**) the procedures for preparing NiO/NiCo_2_O_4_ composites; (**b**) electromagnetic wave attenuation mechanism.

The synthesis process of lotus roots-like NiO/NiCo_2_O_4_ composites is schematically illustrated in Fig. 1a. Flakes structured NiCo-MOFs are firstly synthesized via a hydrothermal method using Co(acac)_2_, Ni(NO_3_)_2_·6H_2_O, and H_2_BDC as precursors. Then, an annealing treatment at 400 °C for 2 h under nitrogen protecting gas flow and 600 °C for 3 h are carried out to convert the NiCo-MOFs into lotus roots-like NiO/NiCo_2_O_4_ hybrids. Figure 1b illustrated the electromagnetic wave attenuation mechanism of the composites. There exists evident interference hardening loss when the matching thickness of absorber meets the geometrical effect, which can be confirmed by the following explanation.

**Figure 2:**
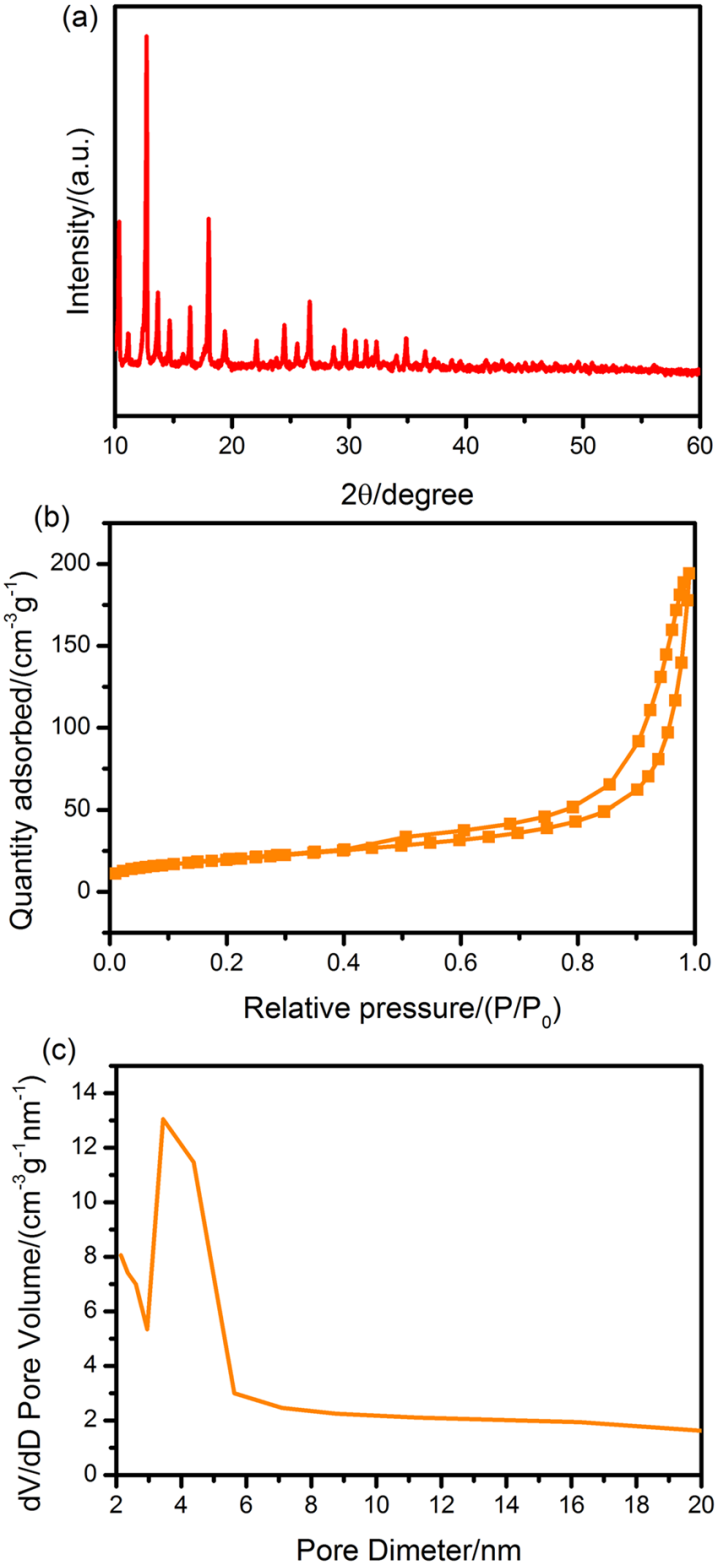
(**a**) XRD pattern; (**b**) N_2_ adsorption-desorption isotherms; (**c**) Pore size distribution plots of the NiCo-MOFs precursor.

In order to study the NiO/NiCo_2_O_4_ composites better, we research the NiCo-MOFs firstly. The powder XRD pattern of the hydrothermal synthesized NiCo-MOF precursor, as shown in Fig. 2a, no residues or contaminants are observed, indicating the high purity of the samples. It is corresponding to the before reports^30–33^. The intensive and sharp peaks of the sample confirm the high crystallinity. The surface areas and pore size distributions of samples investigated by N_2_ adsorption-desorption isotherms are presented in Figs 2b and c, as summarized in Table 1. The pore size distribution curves of the samples show a great deal of disordered porous and a pore size distribution from 2 to 20 nm.

**Table 1:** Surface areas and total pore volumes of NiCo-MOFs precursor and NiO/NiCo_2_O_4_ hybrids.

Sample	S_BET_ (m^2^/g)	S_Langmuir_ (m^2^/g)	V_pore_ (cm^3^/g)
NiCo-MOFs precursor	70.7	150.2	0.30
NiO/NiCo_2_O_4_ hybrid	105.4	240.6	0.15

**Figure 3:**
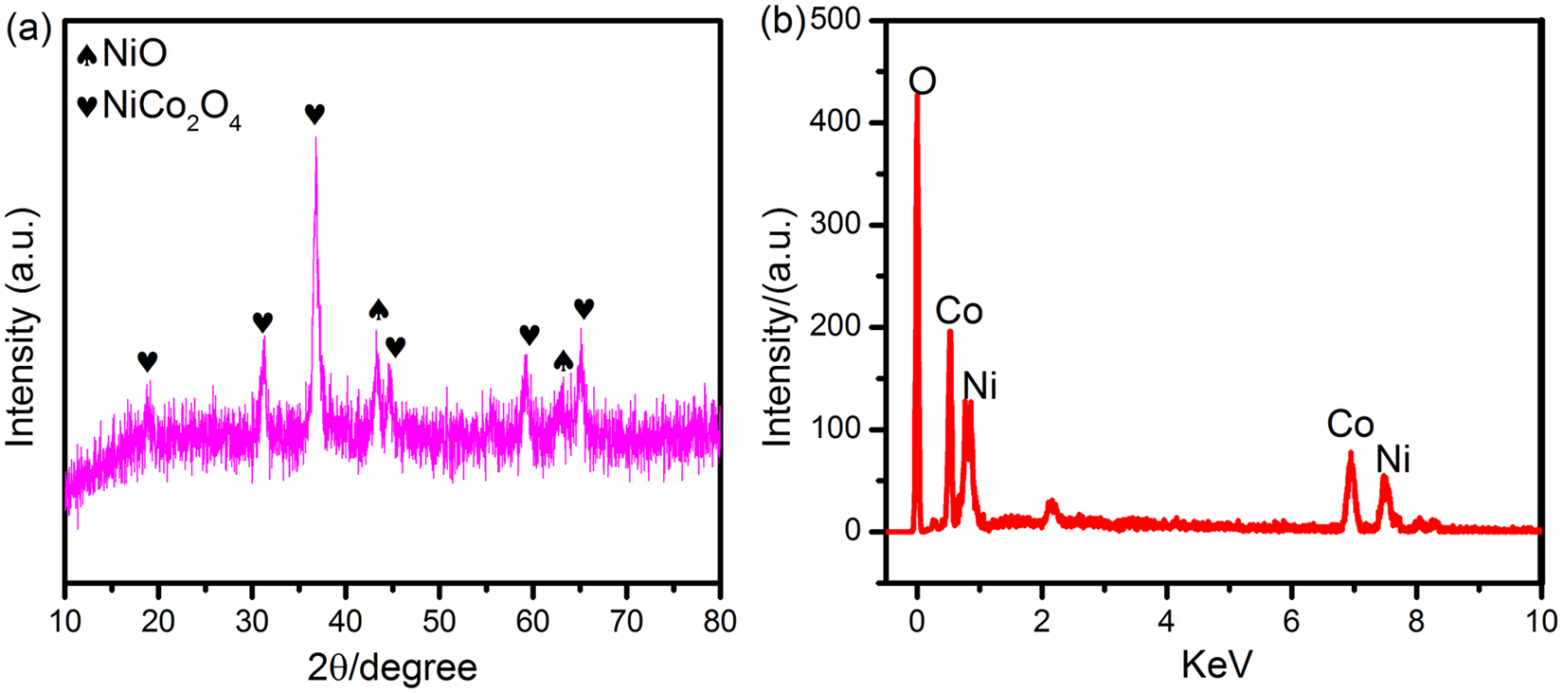
XRD (**a**) and EDS (**b**) patterns of NiO/NiCo_2_O_4_ nanohybrids.

The corresponding powder X-ray diffraction patterns of the annealed samples can provide information on crystallinity and phase components of the synthesized products (Fig. 3a). Diffraction peaks around 43.1° and 62.6° can be respectively indexed as diffractions from the (200) and (220) planes of NiO with a rhombohedral structure^34^. In addition, all of the diffraction peaks at 18.9°, 31.3°, 36.7°, 44.5°, 59.0°, and 64.9° are indexed as the (111), (220), (311), (400), (511), and (440) crystal planes of NiCo_2_O_4_^35, 36^, respectively, in accord with the JCPDS No. 20–0781 (a_0_ = 8.110 Å). No residues or contaminants are observed, indicating the high purity of the samples, moreover, the intensive and sharp peaks of the sample confirm the high crystallinity. EDS analysis reveals that only Co, Ni and O elements existed in the NiO/NiCo_2_O_4_ sample, as shown in Fig. 3b. The atomic ratios of samples tested by ICP (test Co/Ni ratio: 1:1.89) is close to the initial Ni^2+^ and Co^2+^ ratio.

**Figure 4:**
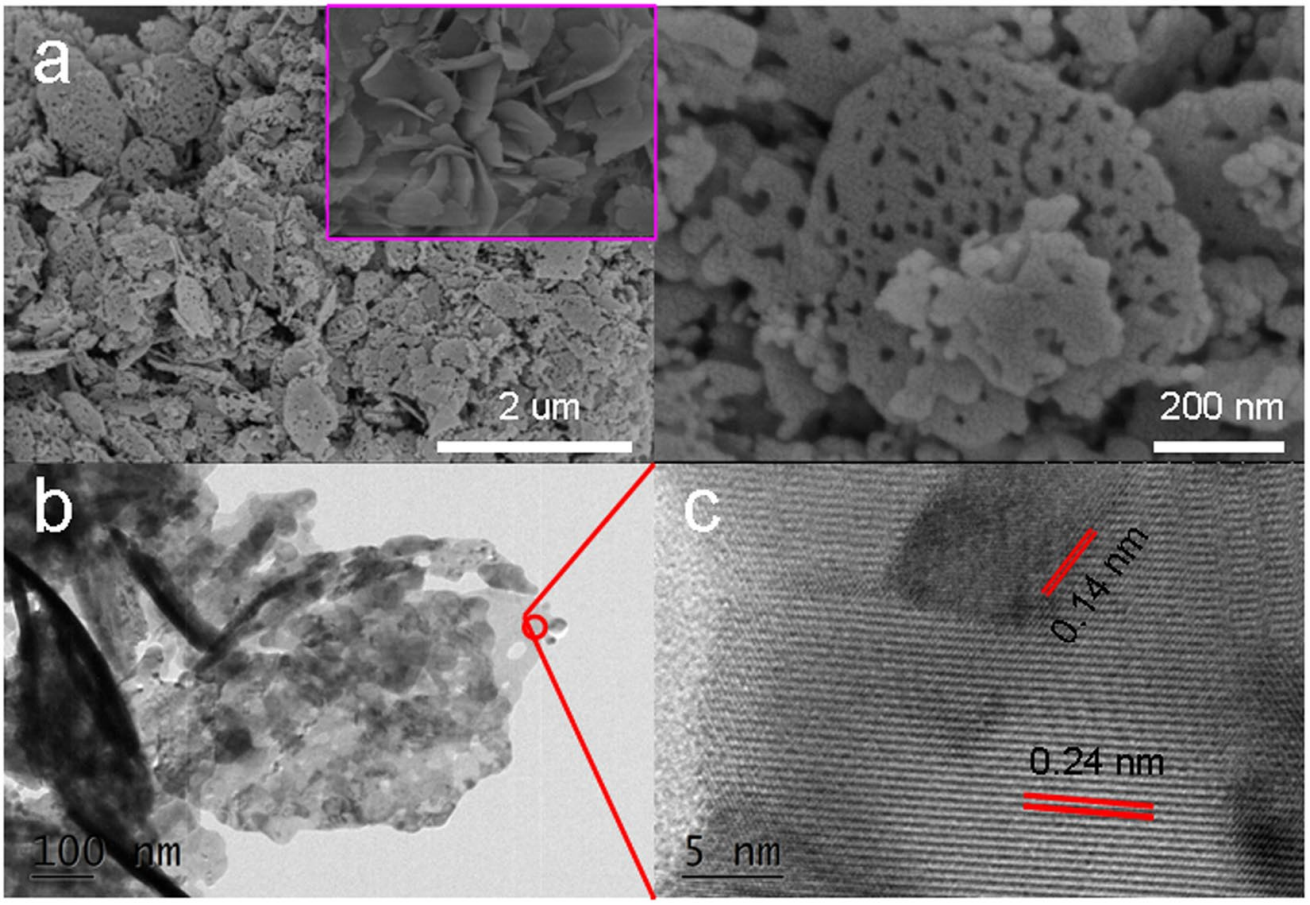
(**a**) FESEM images of NiO/NiCo_2_O_4_ (**a**), insert is the NiCo-MOFs precursor; (**b**) low-magnification TEM image; (**c**) HRTEM image of NiO/NiCo_2_O_4_ nanohybrids.

Representative field-emission scanning electron microscopy (FESEM) and transmission electron microscopy (TEM) images of the precursor are shown in Fig. 4. Figure 4a shows that well-defined uniform NiCo-MOF nanoflakes (insert in Fig. 4a) and lotus roots-like NiO/NiCo_2_O_4_ composites with an average size of around 0.6 μm are obtained. A low TEM image of a single nanoparticle in Fig. 4b revealed that the synthesized product displays a typical lotus roots-like structure with a diameter around of 600 nm, illustrating the flakes from the precursor is well-maintained. Figure 4c depicts HRTEM lattice images on the interface between NiO and NiCo_2_O_4_ sections. The d-spacings of 0.14 nm is in agreement with the (220) plane of NiO, while the d-spacings of 0.24 nm is in well accordance with the (311) plane of NiCo_2_O_4_.

**Figure 5:**
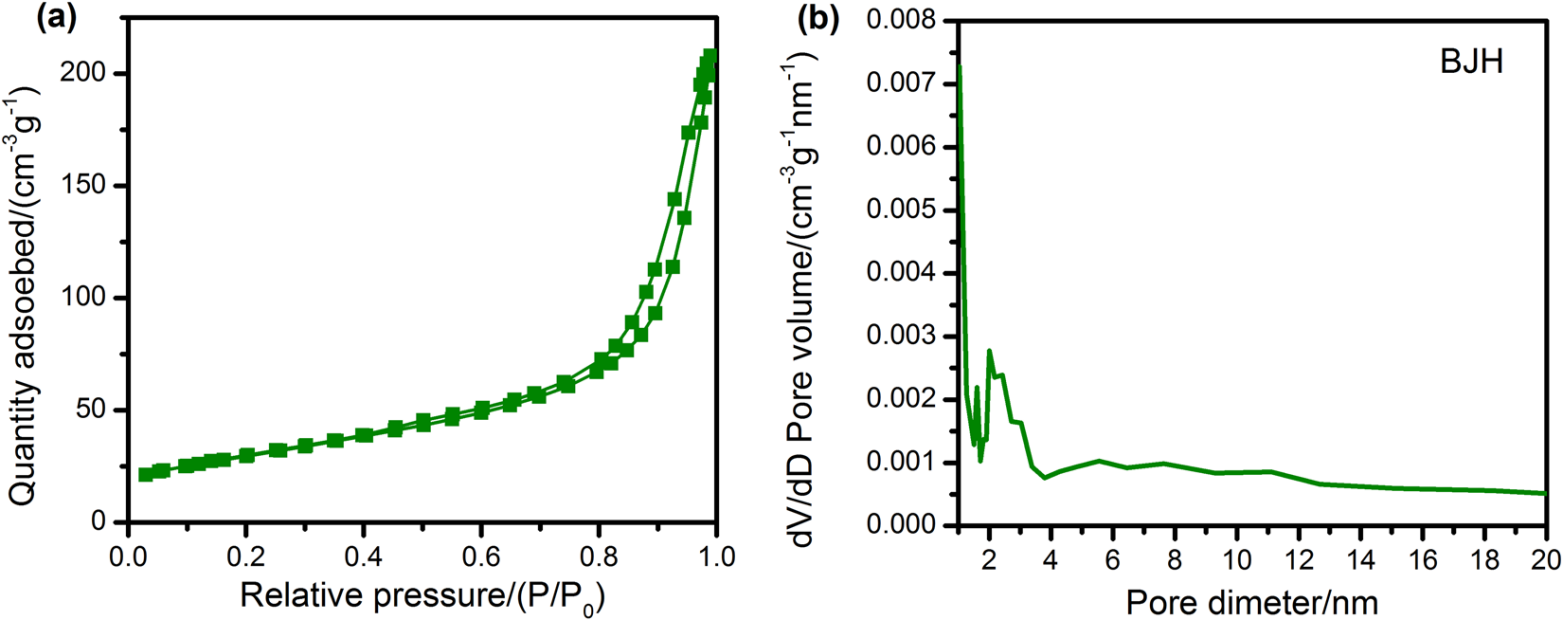
(**a**) N_2_ adsorption-desorption isotherms; (**b**) Pore size distribution plots with BJH of the annealed NiCo-MOFs precursor.

The surface areas and pore size distributions of samples investigated by N_2_ adsorption-desorption isotherms are presented in Fig. 5a and b, as summarized in Table 1. The NiO/NiCo_2_O_4_ hybrids showed high surface area, which indirectly proves a successful approach for reception of the microwave. The pore size distribution curves of the samples show a great deal of disordered porous and pore size distribution from 2 to 20 nm, which may be attributed to the collapse of the well-defined microporous structure of NiCo-MOFs (Fig. 2b and c). Nevertheless, the thermal composites still possess a high surface area and total pore volume, which provide more active sites for reflection and scattering of electromagnetic waves.

## Discussion

**Figure 6:**
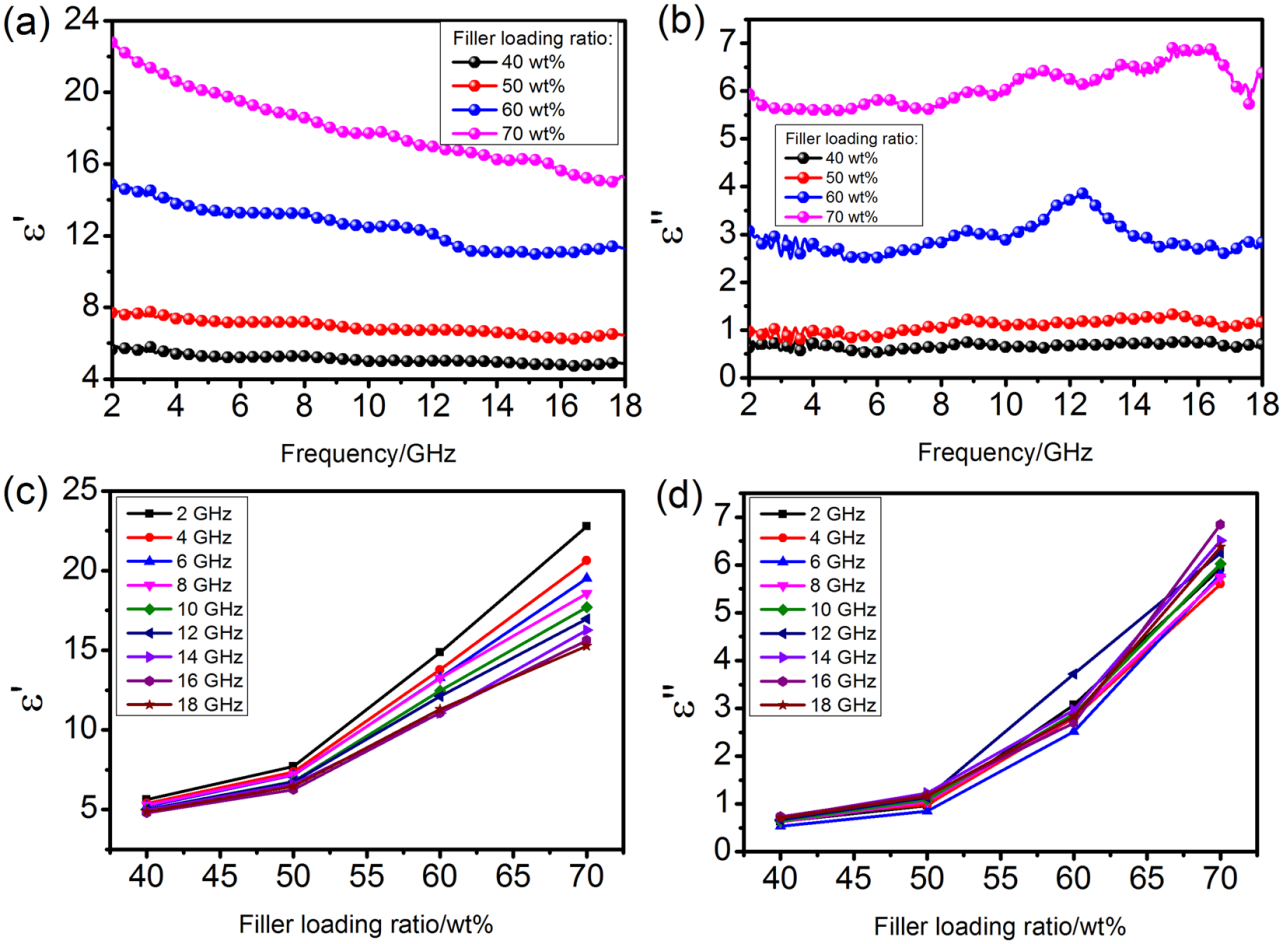
Real part (**a,c**) and imaginary part (**b,d**) of relative complex permittivity of NiO/NiCo_2_O_4_-wax composites with filler loading ranging from 40 wt % to 70 wt %.

Figure 6 shows the complex permittivity of the as-fabricated NiO/NiCo_2_O_4_-wax composites. These composites present typical frequency dependent permittivity, the values of real (*ε′*) permittivity decreases with the frequency in the tested region (Fig. 6a). On the basis of the Debye theory, *ε′* and *ε″* can be described as^37^

1 $$\varepsilon ^{\prime} ={\varepsilon }_{\infty }+({\varepsilon }_{s}-{\varepsilon }_{\infty })/(1+{\omega }^{2}{\tau }^{2})$$

2 $$\varepsilon ^{\prime\prime} =({\varepsilon }_{s}-{\varepsilon }_{\infty })\omega \tau /(1+{\omega }^{2}{\tau }^{2})+{\sigma }_{ac}/{\omega }_{\varepsilon 0}$$

where *ε*_*s*_ is the static permittivity, *ε*_*∞*_ is the relative dielectric permittivity at the high-frequency limit, *ω* is angular frequency, *τ* is polarization relaxation time, *σ*_*ac*_ is the alternative conductivity, and *ε*_0_ is the dielectric constant in vacuum (*ε*_0_ = 8.854 × 10^−12^ F m^−1^). According to Eq. 1, the decrease in *ε′* is attributed to the increase in *ω*. This phenomenon can probably be considered as the polarization relaxation in the lower frequency. With the increase of NiO/NiCo_2_O_4_ loading (from 40 wt % to 70 wt %), significant enhancement was achieved in both *ε′* and imaginary (*ε″*) permittivity (Fig. 6c,d). The increment of *ε*′ may be attributed to the fact that the increasing loading ratio of NiO/NiCo_2_O_4_ improves the dipolar polarization^37, 38^.

The tangent of dielectric loss angle (*δε*) of the material can be expressed as^39^

3 $$\tan \,\delta \varepsilon =\varepsilon ^{\prime\prime} /\varepsilon ^{\prime} $$

**Figure 7:**
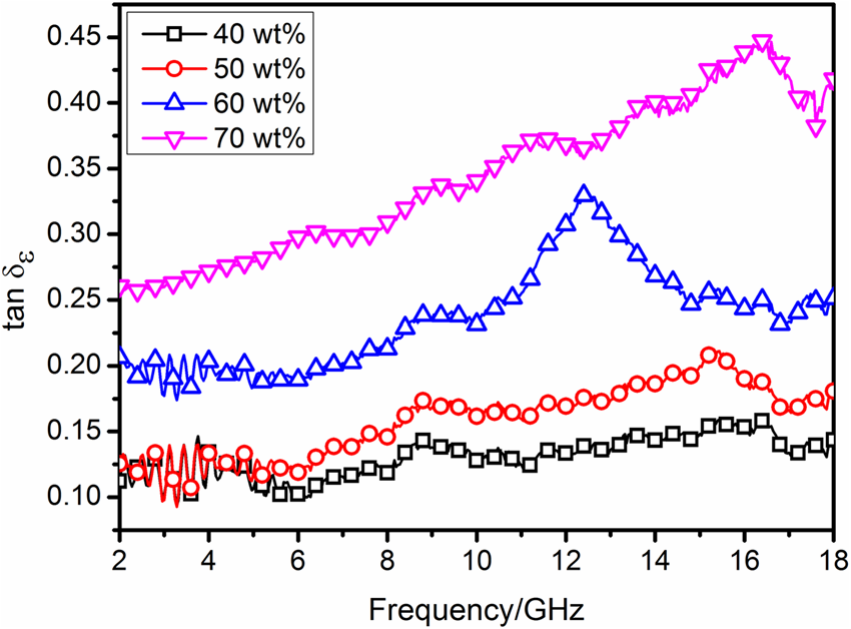
Dielectric loss tangents of NiO/NiCo_2_O_4_-wax composites with filler loading ranging from 40 wt% to 70 wt%.

Figure 7 shows tan *δε* of the composites versus frequency at different loading levels of NiO/NiCo_2_O_4_. In general, the values of *ε″* (Fig. 5B) and tan *δε* both increase with the filler loading ratio, and several relaxation peaks can be found for each curve in the tested frequency range.

When the second part of the Eq. 2 is not taken into account, the relationship between *ε′* and *ε″* can be written as

4 $${(\varepsilon ^{\prime} -({\varepsilon }_{s}+{\varepsilon }_{\infty })/2)}^{2}+{(\varepsilon ^{\prime\prime} )}^{2}={(({\varepsilon }_{s}-{\varepsilon }_{\infty })/2)}^{2}$$

**Figure 8:**
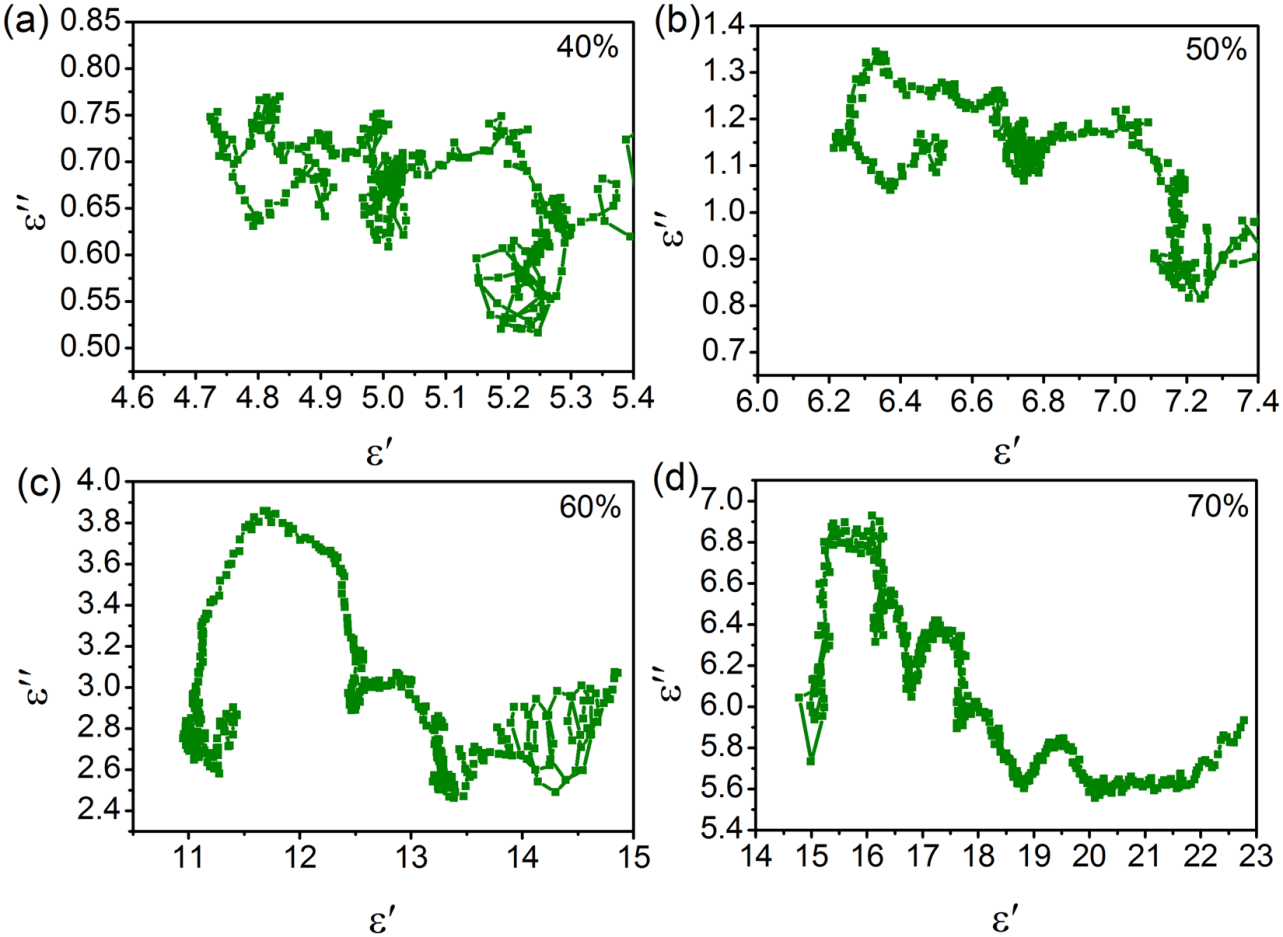
Cole-Cole plots of NiO/NiCo_2_O_4_-wax composites: (**a**) 40 wt %; (**b**) 50 wt %; (**c**) 60 wt %; (**d**) 70 wt %.

It corresponds to a circle centered at ((*ε*_*s*_ + *ε*_*∞*_)/2, 0), which is characteristic for Debye relaxation process. As shown in Fig. 8, each Cole-Cole curve of the NiO/NiCo_2_O_4_-wax composite is very complicated, containing many individual semicircles, due to the multi-relaxations dielectric properties. These multi-relaxations can be well explained by the mechanism proposed by Wu *et al*.^40^. They are supposed to the multiple interfacial polarizations in NiO/NiCo_2_O_4_ hybrids.

Because the frequency range is 2–18 GHz, the source-toshield distance is greater than the free-space wavelength. Thus, the measurements are considered under the condition of far field^41^. According to the transmission line theory^42^, the input impedance (*Z*_*in*_) on the interface can be expressed as Eq. 5.

5 $${Z}_{in}={Z}_{0}{({\mu }_{r}/{\varepsilon }_{r})}^{1/2}\,\tan \,h[j(2\pi fd{({\mu }_{r}{\varepsilon }_{r})}^{1/2}/c)]$$

6 $$RL({\rm{dB}})=20\,\mathrm{log}\,|({Z}_{in}-{Z}_{0})/({Z}_{in}+{Z}_{0})|$$

Where *Z*_*in*_ is the input impedance of the absorber, *f* is the frequency of electromagnetic wave, *d* is the coating thickness of the absorber while *c* is the velocity of electromagnetic wave in free space. *ε*_*r*_ (*ε*_*r*_ = *ε*′*-jε*″) and *µ*_*r*_ (*µ*_*r*_ = *µ*′*-jµ*″) are the complex permittivity and permeability of the absorber. Considering the weak magnetic properties of NiO/NiCo_2_O_4_, *μ*_*r*_ is taken as 1. On the basis of the model of metal backplane, the reflection loss (RL) of a sample is determined by *Z*_0_ and *Z*_*in*_ according to the equation 6^43^. When the RL is lower than −10 dB, more than 90% of the electromagnetic energy is absorbed, implying that this frequency range can be considered as an effective absorption bandwidth.

**Figure 9:**
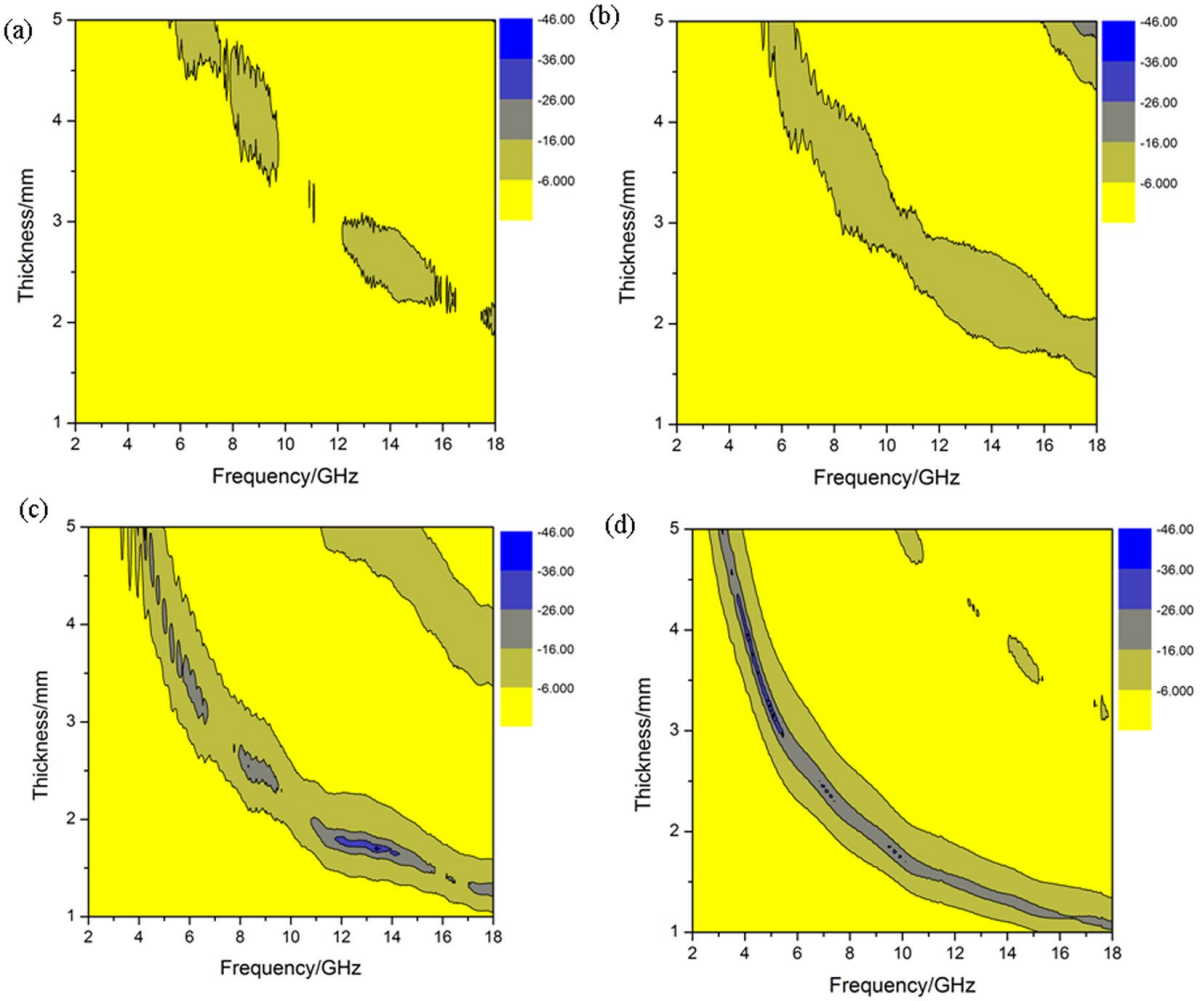
The RL data and effective frequency of NiO/NiCo_2_O_4_-wax composites: (**Aa**) 40 wt %; (**b**) 50 wt %; (**c**) 60 wt %; (**d**) 70 wt %.

**Figure 10:**
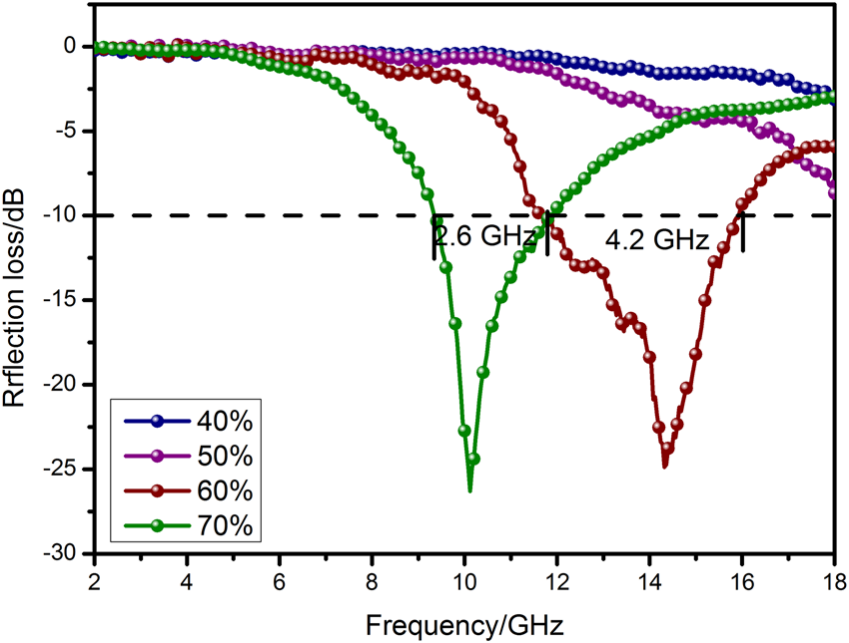
Comparisons of reflection loss values of as-prepared NiO/NiCo_2_O_4_-wax composites.

The effect of the thickness of NiO/NiCo_2_O_4_-wax composite on the electromagnetic wave absorption performance was investigated, and the results are shown in Fig. 9. It can be found that the electromagnetic wave absorption performance improves gradually with the increase of filler loading from 40 to 60 wt% (Fig. 9a–c). Nevertheless, degraded electromagnetic wave absorption performance was observed for the sample with the filler loading ratio of 70.0 wt% (Fig. 9d). According to the fundamental mechanism of electromagnetic wave absorption, the most effective absorption is exhibited when the impedance match between absorber and free space is achieved^37, 44, 45^. The NiO/NiCo_2_O_4_ (60 wt%)-wax composites (Fig. 10) reveal the strong microwave absorption properties at the thickness of 1.6 mm, and the highest effective absorption bandwidth of 4.2 GHz is achieved for the composite at the thicknesses of 1.6 mm (11.8–16 GHz). In addition, the NiO/NiCo_2_O_4_ (70 wt%)-wax composites shows the strongest EM wave absorption with an RL value of −27.1 dB at 10.2 GHz among the four specimens. The thickness of the absorber is a significant factor which influencing the reflection loss value and the frequency of maximum absorption.

**Figure 11:**
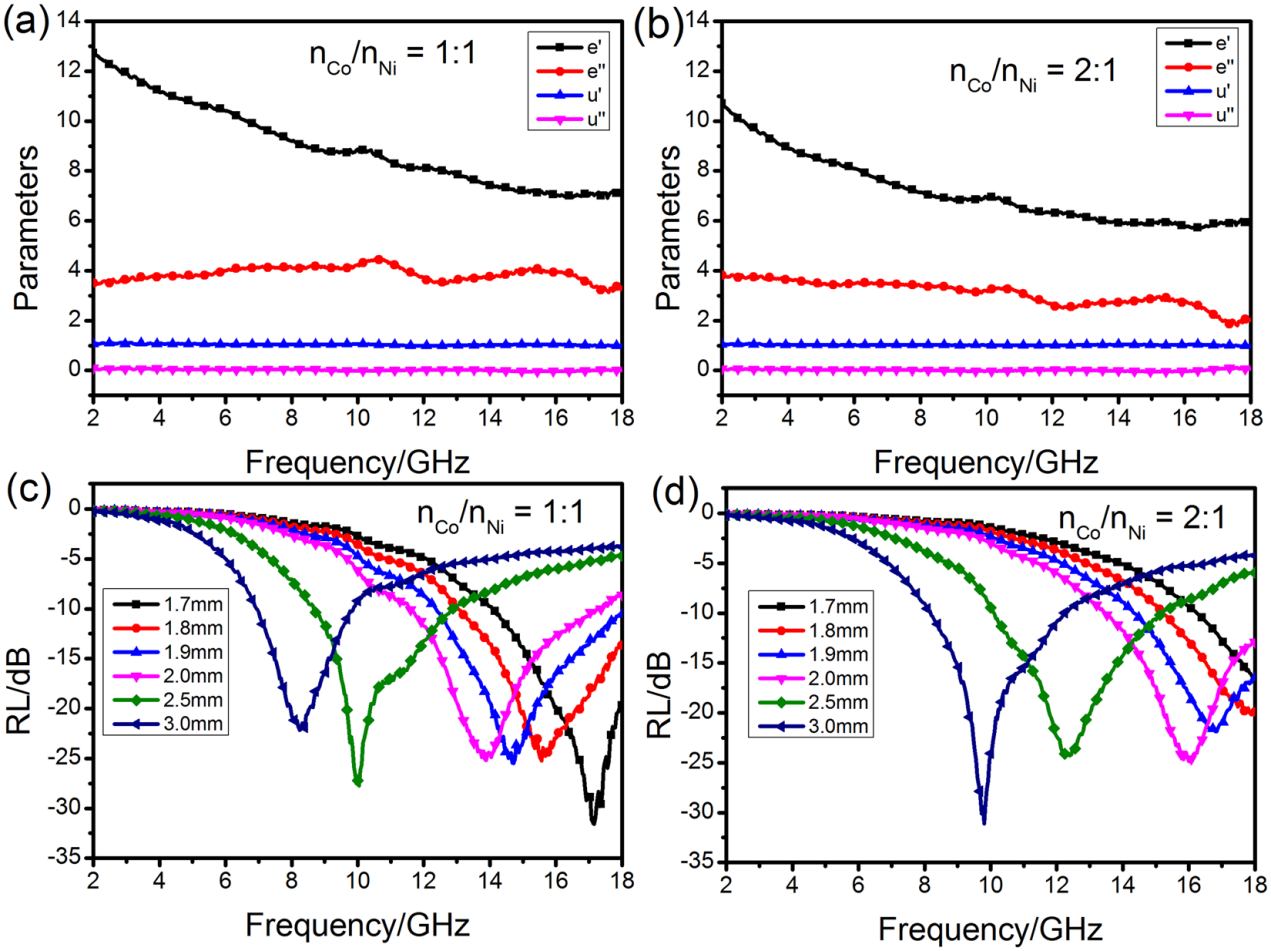
Permittivity and permeability of NiO/NiCo_2_O_4_ composites with different ratio of n_Co_/n_Ni_ = 1:1 (**a**) and n_Co_/n_Ni_ = 2:1 (**b**); RL plots of NiO/NiCo_2_O_4_ composites with different ratio of n_Co_/n_Ni_ (**c** and **d**).

We also investigated the electromagnetic parameters (complex permittivity and permeability) of NiO/NiCo_2_O_4_ composites with different ratio of n_Co_/n_Ni_ to reveal their microwave absorbing properties, shown in Fig. 11a,b. Figure 11a and b shows the *ε′* and *ε″* of complex permittivity in the frequency range of 2–18 GHz. It can be found that both the *ε′* and *ε″* values decrease with increasing frequency in 2–18 GHz, which may be related to a resonance behavior that is reported before^43^. With the increased ratio of n_Ni_, the *ε′* and *ε″* all decreased. When the ratio of n_Co_/n_Ni_ = 1:1, the minimum RL values is −33 dB at 17.1 GHz with a thickness of 1.7 mm (Fig. 11c). Whereas, the ratio of n_Co_/n_Ni_ = 2:1, the minimum RL values is −32 dB at 9.7 GHz with a thickness of 3.0 mm (Fig. 11d). It only reaches −17 dB at 1.7 mm.

**Figure 12:**
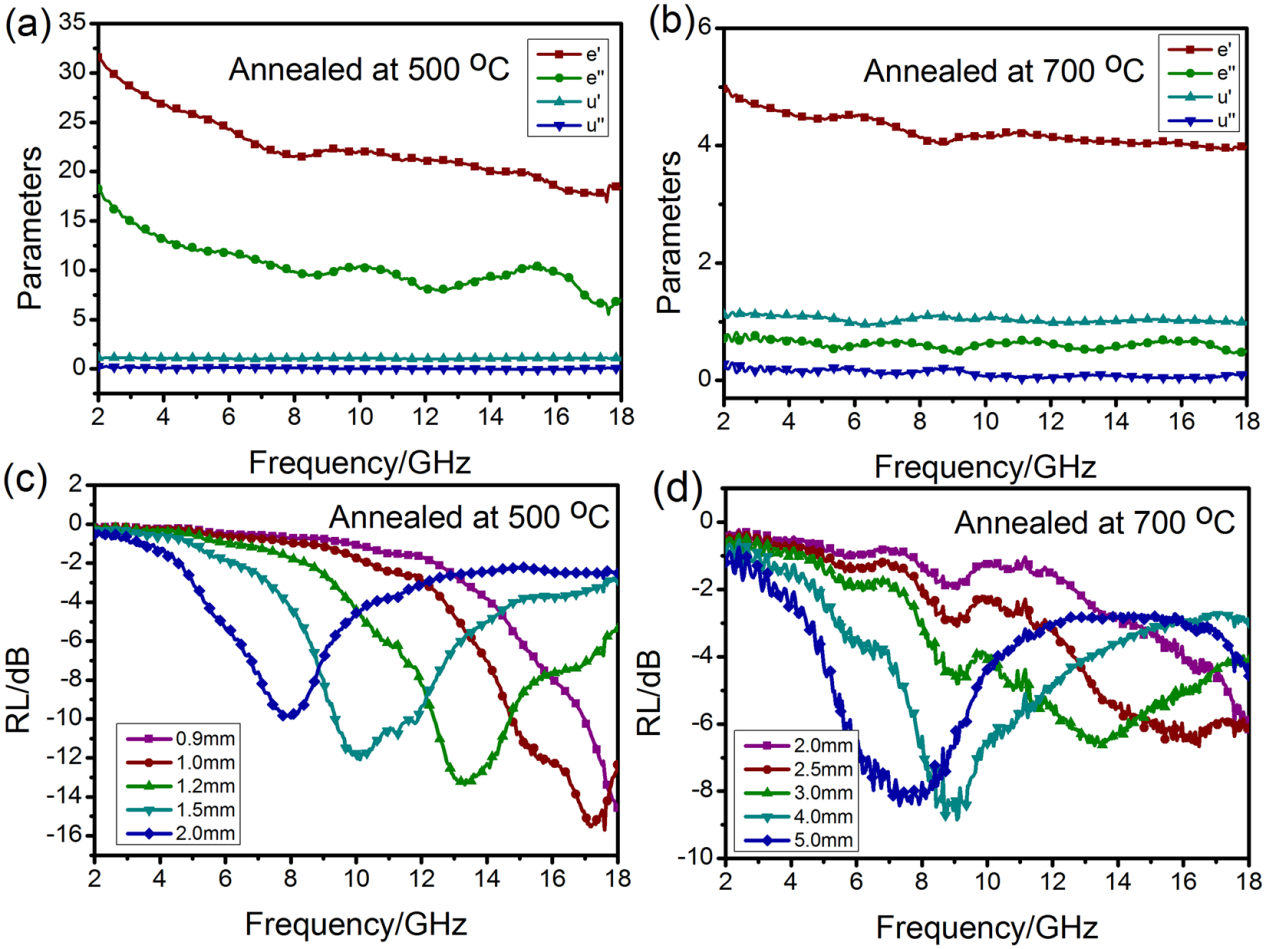
Permittivity and permeability of NiO/NiCo_2_O_4_ composites with different annealing temperature of 500 °C (**a**) and 700 °C (**b**); RL plots of NiO/NiCo_2_O_4_ composites with different annealing temperature (**c** and **d**).

From the Fig. 12a and b, one can find that the *ε′* and *ε″* were all reduced with increasing the annealing temperature. The RL values for calcined samples at 700 °C (Fig. 12d) cannot reach −10 dB within the thickness of 2.0–5.0 mm, indicating that both samples could hardly be used for practical applications. Clearly, the absorption peaks for the calcined sample at 500 °C (Fig. 12c) shift toward a low frequency region as the absorber thickness increases from 0.9 to 2.0 mm. The RL values less than −10 dB are found, moreover, an RL value of 15.3 dB is achieved at 17.2 GHz. The results suggest that the calcined temperature have a huge influence, which is of great interest for the military radar. In conclusion, only in the ratio of n_Co_/n_Ni_ = 1:2 and the annealing temperature of 600 °C can gain the ideal the microwave absorption.

Generally, apart from the magnetic loss and dielectric loss, the microwave can also be absorbed by means of “geometrical effect”^46^. If the thickness of absorber (*t*_*m*_) at the peak frequency (*f*_*m*_) satisfies the equation:

7 $${t}_{m}=nc/(4{f}_{m}{(|{\mu }_{r}||{\varepsilon }_{r}|)}^{1/2})(n=1,3,5\ldots )$$

**Figure 13:**
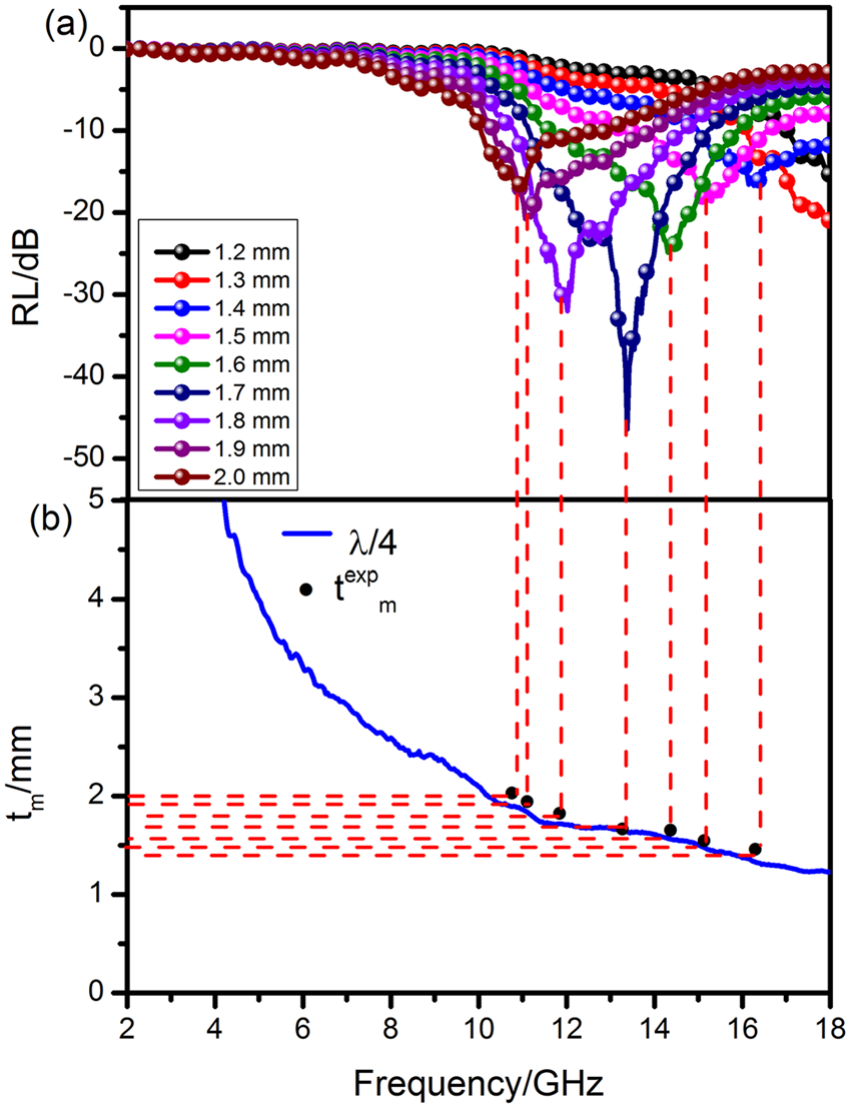
Comparison of various absorber thickness (*t*_*m*_) for the NiO/NiCo_2_O_4_-wax (60 wt %) composites with the simulated thickness under λ/4 conditions at the frequency of maximum RL values (*f*_*m*_).

Where *c* is the velocity of light in the free, *|μ*_*r*_ *|* and *|ε*_*r*_ *|* are the moduli of *μ*_*r*_ and *ε*_*r*_, the incident and reflected waves in the absorbers are out of phase by 180°, bringing about an extinction of each other at the air-absorber interface. To illustrate the reason why the maximum RL value appear at the thickness of 1.7 mm, we conduct the simulations of absorber thickness (*t*_*m*_) at the minimum RL values versus peak frequency (*f*_*m*_) for the NiO/NiCo_2_O_4_-wax (60 wt%) composites under λ/4 conditions^47^, as shown in Fig. 13. The blue curve represents the simulation thickness (t^fit^_m_) at 2–18 GHz using the quarter wavelength principle and the black dots are the experimental matching thickness (t^exp^_m_) at the frequency of maximum RL values (*f*_*m*_). It can be found that the value of t^exp^_m_ at 1.7 mm is well consistent with the simulation t^fit^_m_, while the t^exp^_m_ at other thicknesses deviate from the t^fit^_m_ to a variable extent. Thus, the phenomenon that optimum EM wave absorption activity appears at 1.7 mm can be explained by the geometrical effect. The best EM wave absorption property benefits from the combination of moderate impedance matching character and attenuation loss ability. In addition, interference hardening loss is another important dissipation factor other than the dielectric and magnetic loss and the quarter-wave principle is an effective tool for offering a crucial guide in the thickness design of the microwave absorber.

To the best of our knowledge, the ultimate electromagnetic wave dissipation derives from the comprehensive effect of dielectric and magnetic loss. In general, the integral losses ability is evaluated by the attenuation constant *α*, as expressed in Eq. 8

8 $$\alpha ={2}^{1/2}\Pi f{((\mu ^{\prime\prime} \varepsilon ^{\prime\prime} -\mu ^{\prime} \varepsilon ^{\prime} )+{({(\mu ^{\prime\prime} \varepsilon ^{\prime\prime} -\mu ^{\prime} \varepsilon ^{\prime} )}^{2}+{(\mu ^{\prime} \varepsilon ^{\prime\prime} +\mu ^{\prime\prime} \varepsilon ^{\prime} )}^{2})}^{1/2})}^{1/2}/c$$

**Figure 14:**
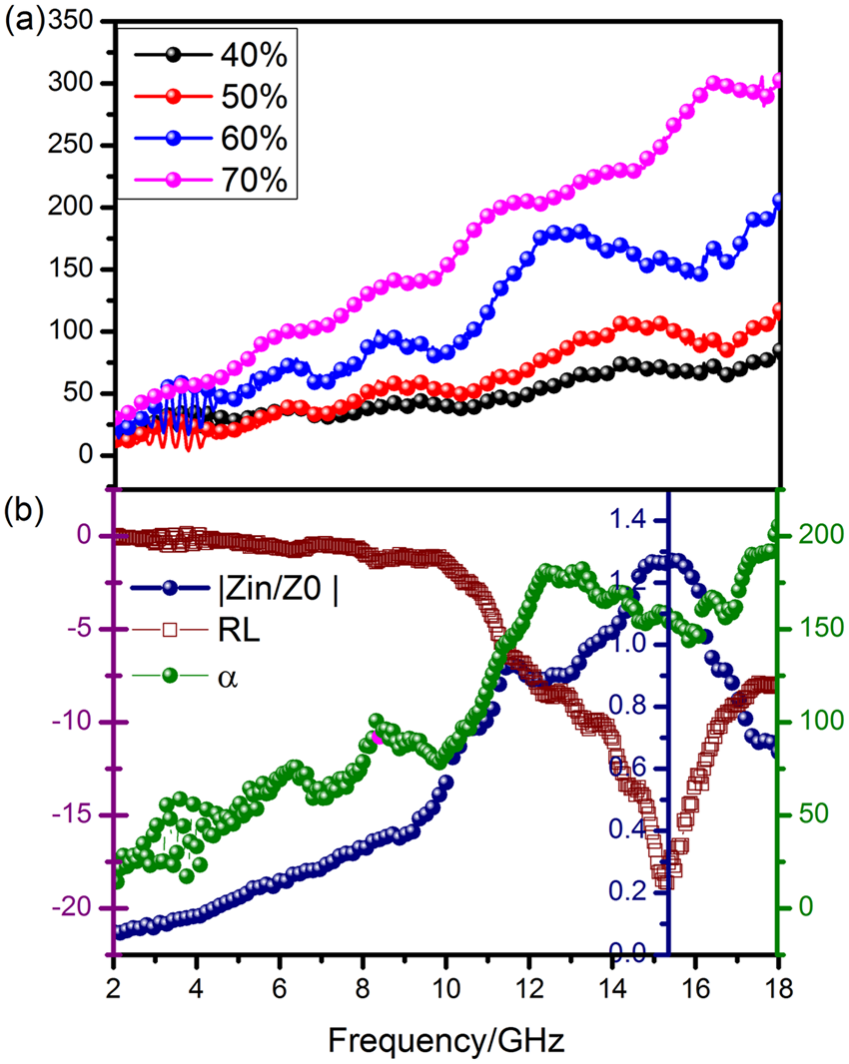
Frequency dependence of attenuation constant (*α*) of all composites (**a**) and RL values, attenuation constant α, and the modulus of normalized input impedance (|*Z*_*in*_ */Z*_0_|) for NiO/NiCo_2_O_4_-wax (60 wt%) composites with 1.5 mm thickness (**b**).

As shown in Fig. 14a, the attenuation capacity of NiO/NiCo_2_O_4_-wax (60 wt%) composites are much higher than NiO/NiCo_2_O_4_-wax (50 wt%) and NiO/NiCo_2_O_4_-wax (40 wt%) samples, indicating the enhanced microwave wastage performance in terms of the electromagnetic wave entering into the interior of the absorbers. However, from an overall perspective, the attenuation ability of sample NiO/NiCo_2_O_4_-wax (70 wt%) is stronger than the sample NiO/NiCo_2_O_4_-wax (60 wt%) which exhibits optimal microwave absorption ability. Therefore, another essential factor (impedance matching) determining the microwave absorbing capacity should be taken into account.

Here, we select NiO/NiCo_2_O_4_-wax (60 wt%) composites with a thickness of 1.5 mm as an example to illustrate the significance of impedance matching on the enhanced microwave absorbing ability of NiO/NiCo_2_O_4_. The value of *Z* = |*Z*_*in*_ */Z*_0_|^48^ was obtained by Eq. 5, where the completely impedance matching will be gained when *Z* = 1. Figure 14b clearly demonstrates the frequency dependence of RL values, attenuation constant *α* and the modulus of normalized input impedance (|*Z*_*in*_ */Z*_0_|) for NiO/NiCo_2_O_4_ (60 wt%) with the thickness of 1.5 mm. When the attenuation constant reaches the maximum value at 18 GHz, the minimum RL can not be obtained and corresponding *Z* is about 0.61. The minimum RL appears when *Z* is close to 1 while the relevant attenuation loss value is only 155 (the maximum attenuation constant is 210). The result gives a reasonable explanation why NiO/NiCo_2_O_4_ (60 wt%) possesses optimal electromagnetic wave absorbing capacity while its dissipation ability is not the most outstanding. It is the impedance matching that acts a critical role for the effective absorbing of microwave^43, 49^. If the impedance matching is poor, strong attenuation loss ability will make no sense for little entered electromagnetic wave^50, 51^. Meanwhile, the results offer a significant reference for the design desired for an ideal microwave absorber. We should give consideration to both impedance matching and attenuation loss ability^52, 53^.

**Figure 15:**
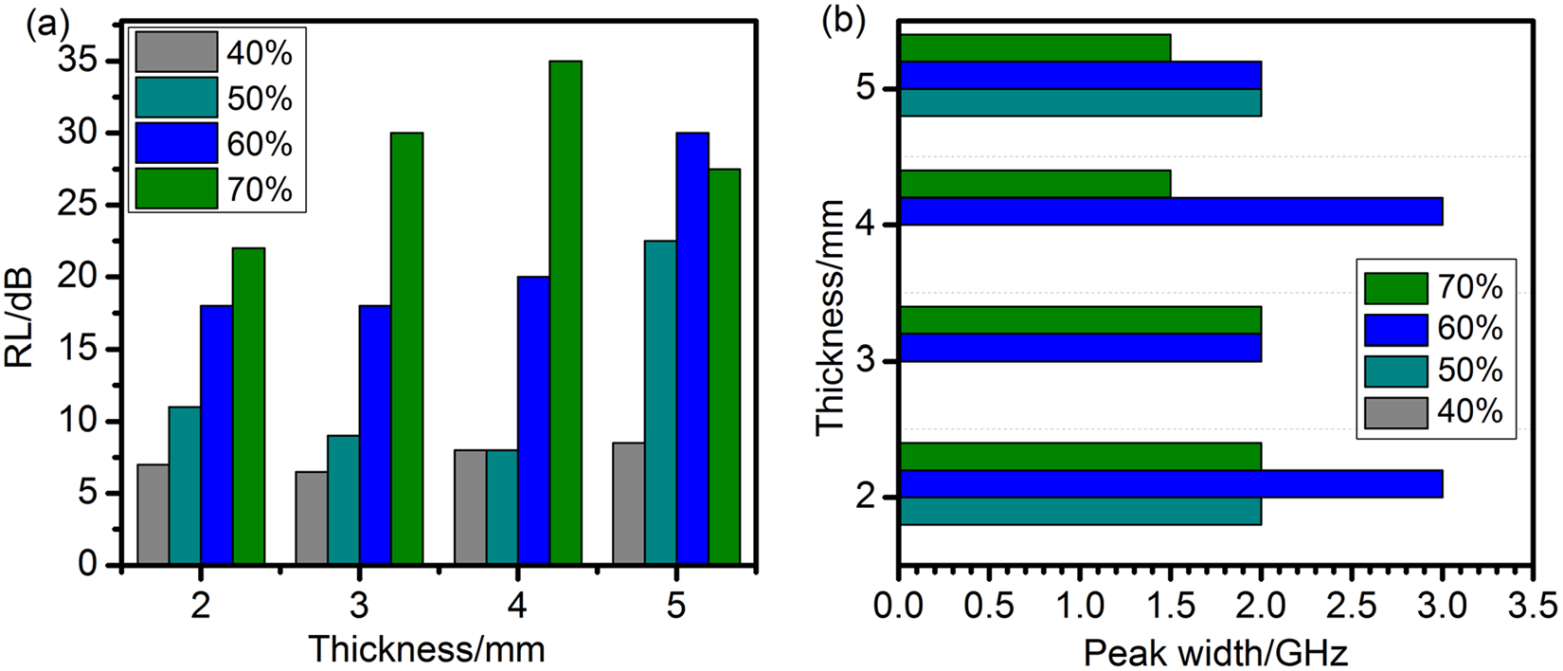
Comparison of (**a**) the reflection loss values and (**b**) peak width (RL < −10 dB) of as-obtained NiO/NiCo_2_O_4_-wax composites at various thickness.

Figure 15a exhibits the comparison of the maximum RL values at the various thicknesses for the samples NiO/NiCo_2_O_4_-wax composites. The NiO/NiCo_2_O_4_-wax (70 and 60 wt%) composites show enormous enhancement of microwave absorption performances at 2–5 mm compared to other samples. The bandwidth (below −10 dB) of the samples NiO/NiCo_2_O_4_-wax composites at 2–5 mm thickness are shown in Fig. 15b. The peak width (RL < −10 dB) of NiO/NiCo_2_O_4_-wax (40 wt %) can not be found at any thickness for its poor microwave absorption property. The NiO/NiCo_2_O_4_-wax (60 wt %) composites exhibit excellent microwave absorption properties in wide frequency scopes. In addition, the sample NiO/NiCo_2_O_4_-wax (60 wt %) has a particularly wide bandwidth of 3.1 GHz at 2 mm. Obviously, the NiO/NiCo_2_O_4_-wax (60 wt %) composites possess the advantages of strong microwave absorption performances and broad absorption bandwidths at a relatively thickness.

## Conclusions

In summary, hybrids of NiO/NiCo_2_O_4_ were prepared and their electromagnetic wave absorption performance was investigated for the first time. The obtained NiO/NiCo_2_O_4_ composites consist of large lotus roots-like plates and exhibits superior electromagnetic wave absorption performance with high efficiency and broad bandwidth at thin thicknesses and low filler loadings. Impressively, an effective bandwidth of 4.2 GHz was observed for a wax-based sample containing 60 wt % NiO/NiCo_2_O_4_ hybrids with a thickness of 1.6 mm. The highest reflection loss of a sample with the thickness of 1.7 mm reached −47 dB at 13.4 GHz. Taking the low cost and high stability into account, we think the hybrids of NiO/NiCo_2_O_4_ are promising electromagnetic wave absorbers and deserve further detailed investigations.

## Method

### Preparation of NiCo Metal-Organic Frameworks Precursor

All of the chemicals in this work were used without further purification. In a typical procedure, cobalt(II) acetylacetonate (Co(acac)_2_, 200 mg), Ni(NO_3_)_2_·6H_2_O (108 mg), 1,4-benzenedicarboxylic acid (H_2_BDC, 24 mg), and poly (vinylpyrrolidone) (PVP; MW = 30000, 500 mg) were dissolved in N,N-dimethylformamide (DMF)-ethanol mixture (5:3 (v/v)) under magnetic stirring at room temperature to form a homogeneous solution. Then the resulted homogeneous solution was transferred to a Teflon-lined stainless-steel autoclave. The sealed vessel was heated to 150 °C, kept there for 12 h, and then cooled to room temperature. The green NiCo-MOFs precursors were obtained after centrifugation and washing with DMF and ethanol for several times.

### Preparation of lotus roots-like NiO/NiCo_2_O_4_ composites

The powder of NiCo-MOFs precursor was placed in a tube furnace and then heated to 400 °C for 1 h with a ramp of 2 °C/min under nitrogen gas flows. After that, the nitrogen gas was switched off, and the furnace was still kept in air at 400 °C for 1 h. In order to obtain NiO/NiCo_2_O_4_, the temperature was heated to 600 °C with a ramp of 5 °C/min and kept in air for 3 h.

### Characterization

The crystal structures of the asprepared materials were characterized by Rigaku D/Max-Rb diffractometer equipped with Cu K*a* radiation (*λ* = 1.5406 Å). The morphologies and structure were observed by SU-70 field-emission scanning electron microscopy (FESEM) and transmission electron microscopy (JEM-2100) at an acceleration voltage of 200 kV. Nitrogen adsorption-desorption isotherms were measured at 77 K using Gold APP Vsorb 2800 P surface area and porosity 60 analyzer. The atomic ratio of Ni and Co is measured by inductively coupled plasma (ICP, Optimal 5300DV).

### Electromagnetic parameters tests

The S parameters including S11, S12, S21 and S22 will be performed by an Agilent PNA N5224A vector network analyzer using the coaxial-line method which the samples were prepared by homogeneously mixing the paraffin wax and sample (mass ratio: 40:60, 50:50, 60:40, 70:30) and then pressing into toroidal-shaped samples (Φ_out_:7.0 mm, Φ_in_:3.04 mm). Subsequently, a software which has been installed in Agilent PNA can deal with the *ε*′, *ε*″, *μ*′, *μ*″ values.
